# Topological holographic quench dynamics in a synthetic frequency dimension

**DOI:** 10.1038/s41377-021-00646-y

**Published:** 2021-10-07

**Authors:** Danying Yu, Bo Peng, Xianfeng Chen, Xiong-Jun Liu, Luqi Yuan

**Affiliations:** 1 State Key Laboratory of Advanced Optical Communication Systems and Networks, School of Physics and Astronomy, Shanghai Jiao Tong University, 200240 Shanghai, China; 2 Shanghai Research Center for Quantum Sciences, 201315 Shanghai, China; 3 Jinan Institute of Quantum Technology, 250101 Jinan, China; 4 Collaborative Innovation Center of Light Manipulations and Applications, Shandong Normal University, 250358 Jinan, China; 5 International Center for Quantum Materials and School of Physics, Peking University, 100871 Beijing, China; 6 Shenzhen Institute for Quantum Science and Engineering, Southern University of Science and Technology, 518055 Shenzhen, China

**Keywords:** Optics and photonics, Physics

## Abstract

The notion of topological phases extended to dynamical systems stimulates extensive studies, of which the characterization of nonequilibrium topological invariants is a central issue and usually necessitates the information of quantum dynamics in both the time and momentum dimensions. Here, we propose the topological holographic quench dynamics in synthetic dimension, and also show it provides a highly efficient scheme to characterize photonic topological phases. A pseudospin model is constructed with ring resonators in a synthetic lattice formed by frequencies of light, and the quench dynamics is induced by initializing a trivial state, which evolves under a topological Hamiltonian. Our key prediction is that the complete topological information of the Hamiltonian is encoded in quench dynamics solely in the time dimension, and is further mapped to lower-dimensional space, manifesting the holographic features of the dynamics. In particular, two fundamental time scales emerge in the dynamical evolution, with one mimicking the topological band on the momentum dimension and the other characterizing the *residue* time evolution of the state after the quench. For this, a universal duality between the quench dynamics and the equilibrium topological phase of the spin model is obtained in the time dimension by extracting information from the field evolution dynamics in modulated ring systems in simulations. This work also shows that the photonic synthetic frequency dimension provides an efficient and powerful way to explore the topological nonequilibrium dynamics.

## Abstract (web summary)

Topological holographic quench dynamics in a synthetic frequency dimension studied in modulated ring system shows the universal bulk-surface duality with complete topological information being encoded in the single time variable.

## Graphical abstract

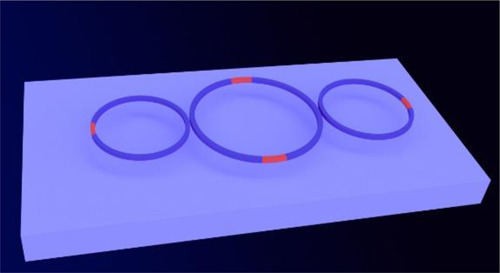

## Introduction

The discovery of topological quantum phases has revolutionized the understanding of the fundamental phases of quantum matter and ignited extensive research in condensed matter physics over the past decades^1–5^. In addition to the great progress made for equilibrium phases, the nonequilibrium quantum dynamics can exhibit exotic behaviors^6,7^, and the notion of topological phases has been extended to far-from-equilibrium dynamical systems, with novel topological physics being uncovered, such as the anomalous topological states in Floquet systems^8–14^ and dynamical topology emerging in quantum quenches^15–29^. In particular, a universal dynamical bulk-surface correspondence was predicted when quenching a system across topological transition^14,30–32^, showing that the bulk topology of an equilibrium topological phase has a one-to-one correspondence to quench-induced dynamical topological patterns emerging on the lower-dimensional momentum subspaces called band inversion surfaces (BISs). The dynamical bulk-surface correspondence connects the equilibrium topological phases with far-from-equilibrium quantum dynamics, which was further extended to correlated system^33^, high-order regimes^34,35^, and to generic nonadiabatic quenches^36^. This opens the way to characterize equilibrium topological phases by nonequilibrium quench dynamics, and inversely, to classify nonequilibrium quantum dynamics by topological theory, with the experimental studies having been widely reported recently^37–44^. The nonequilibrium topological invariants are typically defined via time dimension and momentum space, and their characterization naturally necessitates the information of quantum dynamics in both the time and spatial dimensions.

In order to go beyond the spatial degree of freedom and capture the extensive information of quantum dynamics, the synthetic dimensions^45–47^ were proposed and opened an intriguing avenue towards the quantum simulation of exotic topological physics beyond physical dimensions^48–50^. Following the numerous theoretical proposals on synthetic dimensions using a different degree of freedoms such as the frequency or the orbital angular momentum of light^51–54^, and the hyperfine levels of atoms^55^, experiments have been recently performed to demonstrate the two-dimensional topological insulator^56^ and the Hall ladder^57^ in the synthetic space, where the effective magnetic field for photons is generated, and visualize the edge states^58,59^. Further, the high-dimensional physics can be studied in a photonic platform with lower dimensionality^53,60–64^. More recently, the experimental platforms for generating the synthetic dimension along the frequency axis have also been proposed and demonstrated using the ring resonator^52,53,57,65,66^, in which the photonic modes at equally spanned frequencies are coupled through the dynamic modulation. In this system, the band structure in the synthetic dimension can be measured in the static steady-state regime in the experiment^65^. On the other hand, with the synthetic dimensions the novel optical phenomena and applications have been proposed, including the realizations of unidirectional frequency translation^67^, pulse narrowing^68^, active photon storage^69^, and topological laser^70^. Therefore, synthetic dimensions in photonics not only provide a unique way to simulate quench dynamics but also can find many novel applications for light. In particular, the reciprocal dimension of the frequency axis of light is the time dimension, which naturally arises the question: how to understand the quench dynamics in the synthetic frequency dimension. Successful answering of this question can also trigger further studies of quench dynamics of the complicated model in higher dimensions on modulated ring systems.

In this article, we combine the concepts of dynamical classification and synthetic dimension and propose a highly efficient scheme to characterize topological phases by holographic quench dynamics. The topological holographic quench dynamics refer to a procedure of the quench dynamics simulated by encoding the complete topological information of light fields in a time dimension, which is further mapped to a lower-dimensional subspace through the bulk-surface duality. We construct a one-dimensional pseudospin model in a photonic synthetic lattice in the frequency domain and investigate the quench dynamics by initializing a trivial phase, which evolves under a topological Hamiltonian. We show that the full dynamical evolution is featured by two fundamental time scales, with which the quench dynamics exhibit universal topological patterns. In particular, one time scale mimics the Bloch momenta of the topological band and the other (i.e., the round-trip number of light circulating inside rings) characterizes the *residue* time evolution after the quench. By extracting information of the field onto two time scales in simulations, we find the dynamical topological patterns obtained on BISs, which render an emergent dynamical bulk-surface duality and provide a holographic characterization of the topological spin model, with the complete information being encoded in the single variable, i.e., the time evolution, in sharp contrast to the conventional nonequilibrium topological states. The dynamical topology is robust against disorders and has high experimental feasibility. This work shows advantages in exploring the topological phases with holographic quench dynamics in the synthetic dimensions and provides an insight into classifying the far-from-equilibrium dynamics with nontrivial topology based on the synthetic photonic crystals.

## Results

### Model

**Fig. 1:**
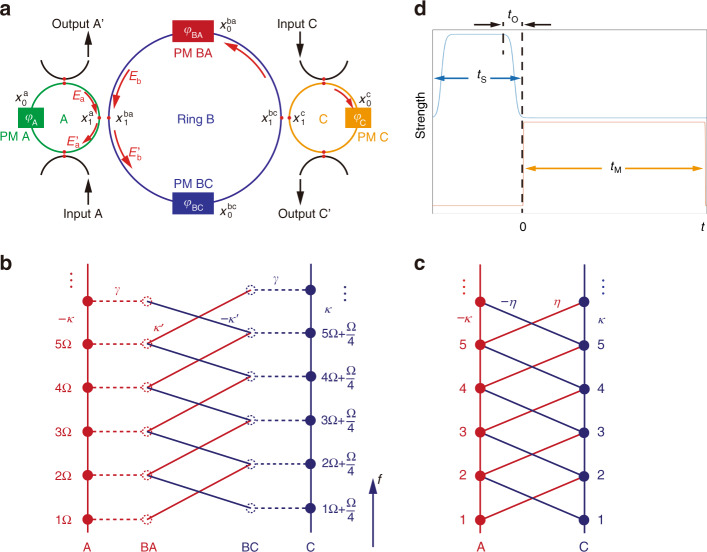
Schematics of the model. **a** A schematic design of the ring-resonator system with phase modulators. External waveguides are used to input (output) signal. **b** The diagram shows couplings between modes ${{{\mathrm{A}}}}_m$ (red solid dot) and ${{{\mathrm{BA}}}}_m$ (red dashed dot) at frequencies $\omega _{{{{\mathrm{A}}}},m} = m{{\Omega }}$ and modes ${{{\mathrm{C}}}}_m$ (blue solid dot) and ${{{\mathrm{BC}}}}_m$ (blue dashed dot) at frequencies $\omega _{{{{\mathrm{C}}}},m} = m{{\Omega }} + {{\Omega }}/4$. Modulators induce nearest-neighbor couplings (solid line) between nearby modes along the frequency axis of light; modes in different rings are coupled through the evanescent wave (dashed line). Red (blue) solid lines denote couplings induced by modulator PM BA (BC). For the nontrivial case, $\phi _{{{\mathrm{A}}}} = \phi _{{{{\mathrm{BC}}}}} = \pi$ and $\phi _{{{\mathrm{C}}}} = \phi _{{{{\mathrm{BA}}}}} = 0$ in modulators give negative and positive couplings, respectively. **c** The effective tight-binding model of the pseudospin lattice in the nontrivial case ($\phi = \pi$). **d** Time sequences of input source ($t_{{{\mathrm{S}}}}$) and modulation ($t_{{{\mathrm{M}}}}$) in simulations. $t_{{{\mathrm{O}}}}$ is the turn-on/turn-off time

We start with illustrating our idea of using ring resonators under dynamic modulations to artificially engineer a tight-binding lattice of pseudospin states along the frequency axis of light. As shown in Fig. 1a, the system under the study in this work contains three ring resonators, with each hosting a set of resonant frequency modes. Two of the resonators (A and C) will be used to mimic a pseudospin-1/2 system. Let the group velocity dispersion be zero in the waveguide that constructs the ring. We set ring A such that it supports a set of resonant modes at frequencies $\omega _{{{{\mathrm{A}}}},m} = m{{\Omega }}$, which refer to a general carrier optical frequency $\omega _0 \gg \Omega$ that we omit for simplicity, where $m$ is an integer and ${{\Omega }} = 2\pi c/Ln_{{{\mathrm{g}}}}$ is the free spectral range of the ring. Here, $c$ is the speed of light, $L$ is the circumference of ring A, and $n_{{{\mathrm{g}}}}$ is the effective refractive index. The resonant modes in the ring C with the same circumference $L$ have frequencies $\omega _{{{{\mathrm{C}}}},m} = m{{\Omega }} + {{\Omega }}/4$, which refer to $\omega _0$. We use ring B with the circumference $4L$ to serve as an auxiliary ring^71^. Ring B has shifted resonant modes at frequencies $\omega _{{{{\mathrm{B}}}},m} = m{{\Omega }}/4 + {{\Omega }}/8$, which refer to $\omega _0$. Hence, although there are evanescent couplings between nearby rings, the field resonantly circling inside ring A(C) is not resonant in the auxiliary ring B.

The couplings between modes in the ring resonators are engineered by properly setting the phase modulators. We place one phase modulator [labeled as PM A(C) in Fig. 1a] inside ring A(C). The light that transmits through the modulator in ring A(C) undergoes dynamical modulation with the transmission coefficient as^72^:

1 $$T_{{{{\mathrm{A(C)}}}}} = e^{i\kappa \,{{{\mathrm{cos}}}}[{{\Omega }}_0t + \phi _{{{{\mathrm{A}}}}({{{\mathrm{C}}}})}]}$$

where $\kappa$ is the modulation strength, ${{\Omega }}_0$ is the modulation frequency, and $\phi _{{{{\mathrm{A}}}}({{{\mathrm{C}}}})}$ is the modulation phase in the modulator PM A(C). We consider the resonant modulation, i.e., ${{\Omega }}_0 = {{\Omega }}$, so each modulator couples the nearest-neighbor resonant modes in two rings in the first-order approximation. Ring B contains two phase modulators, which are labeled as PM BA and PM BC with the corresponding transmission coefficients $T_1$ and $T_2$:

2 $$T_{1( 2 )} = e^{i\kappa^{\prime}\,{{{\mathrm{cos}}}}({{\Omega }}_{1( 2 )}t + \phi _{{{{\mathrm{BA}}}}({{{\mathrm{BC}}}})})}$$

where $\kappa^{\prime}$ is the modulation strength, ${{\Omega }}_{1,2}$ are the modulation frequencies, and $\phi _{{{{\mathrm{BA}}}}}$ and $\phi _{{{{\mathrm{BC}}}}}$ are modulation phases in PM BA and PM BC, respectively. We set ${{\Omega }}_1 = 5{{\Omega }}/4$ so that the field component at the frequency $\omega _{{{{\mathrm{A}}}},m}$ couples with the component at $\omega _{{{{\mathrm{C}}}},m + 1}$. Similarly, for ${{\Omega }}_2 = 3{{\Omega }}/4$, the component at $\omega _{{{{\mathrm{A}}}},m + 1}$ couples with the component at $\omega _{{{{\mathrm{C}}}},m}$.

The pseudospin-1/2 system is realized by modulating the resonator couplings. The field in ring A(C) is coupled with the field in ring B through the evanescent wave. The corresponding coupling equation is described by the coupling matrix between input electric field amplitudes *E*_a_, *E*_b_ and output amplitudes $E^{\prime}_{{{\mathrm{a}}}}$ and $E^{\prime}_{{{\mathrm{b}}}}$ labeled in Fig. 1a:

3 $$\left( {\begin{array}{*{20}{c}} {E^{\prime}_{{{\mathrm{a}}}}} \\ {E^{\prime}_{{{\mathrm{b}}}}} \end{array}} \right) = \left(\begin{array}{*{20}{c}}{\sqrt{1-\gamma^2}} & {-i\gamma} \\{-i\gamma} & {\sqrt{1-\gamma^2}}\end{array}\right)\left( {\begin{array}{*{20}{c}} {E_{{{\mathrm{a}}}}} \\ {E_{{{\mathrm{b}}}}} \end{array}} \right)$$

Here, $\gamma$ is the coupling strength. The coupling matrix between ring B and ring C follows the same expression. With the above ingredients, we can map the setting to the diagram described in Fig. 1b, which gives our lattice model as shown below. Here, the description of external waveguides used for the input source and output detections in simulations are not included.

The coupling between resonant modes in rings A and C are mediated by ring B, as illustrated in Fig. 1b. The physics is described below. The energies of the resonant modes ${{{\mathrm{A}}}}_m$ leak into the temporary nonresonant component (labeled as ${{{\mathrm{BA}}}}_m$) in ring B, which may decay quickly. However, the modulations characterized in Eq. (2) in ring B convert the energies of these components ${{{\mathrm{BA}}}}_m$ to other nonresonant components (labeled as ${{{\mathrm{BC}}}}_m$), and the latter components are transferred to resonant modes ${{{\mathrm{C}}}}_m$ in ring C. Hence, the couplings to the auxiliary ring B serve as an intermediate process that mediates a second-order coupling between resonant modes ${{{\mathrm{A}}}}_m$ and ${{{\mathrm{C}}}}_m$. This process mimics the second-order Raman process between two states through virtual transitions to an intermediate state in quantum mechanics. On the other hand, the resonant modes with frequencies $\omega _{{{{\mathrm{A}}}},m}$ (labeled as ${{{\mathrm{A}}}}_m$) in ring A couple between each other through the dynamic modulation characterized in Eq. (1), forming a synthetic lattice for A itself in the frequency dimension, similar to resonant modes ${{{\mathrm{C}}}}_m$ in ring C.

We now turn to the effective model of the coupled ring system. The modulations including modulation phases inside rings have high tunability^71^. We choose modulation phases to be either $0$ or $\pi$. For example, we can set $\phi \equiv \phi _{{{\mathrm{A}}}} = \phi _{{{{\mathrm{BC}}}}} = \pi$, which gives the corresponding negative coupling, or $\phi _{{{\mathrm{C}}}} = \phi _{{{{\mathrm{BA}}}}} = 0$, which gives the positive corresponding coupling. By analyzing the field changes of each frequency component at specific positions inside ring systems after it propagating each full circle, one can obtain effective temporal differential coupled-mode equations and hence obtain effective Hamiltonian under the first-order perturbation^67,73^. Such Hamiltonian of the system reads

4 $$\begin{array}{l}H = \mathop {\sum }\limits_m \left( {\omega _0 + \omega _{{\mathrm{A}},m}} \right)a_m^{\dagger} a_m + \left( {\omega _0 + \omega _{{\mathrm{C}},m}} \right)c_m^{\dagger} c_m\\ \quad \quad+ \, \kappa _{{\mathrm{TB}}}\left[ {\cos \left( {\Omega t + \phi } \right)\left( {a_m^{\dagger} a_{m + 1} + a_{m + 1}^{\dagger} a_m} \right)} \right.\\ \quad \quad \left. { + \,\cos \left( {\Omega t} \right)\left( {c_m^{\dagger} c_{m + 1} + c_{m + 1}^{\dagger} c_m} \right)} \right]\\ \quad \quad+\, \eta _{{\mathrm{TB}}}\left[ {\cos \left( {5\Omega t/4} \right)\left( {a_m^{\dagger} c_{m + 1} + c_{m + 1}^{\dagger} a_m} \right)} \right.\\ \quad \quad \left. { + \, \cos \left( {3\Omega t/4 + \phi } \right)\left( {c_m^{\dagger} a_{m + 1} + a_{m + 1}^{\dagger} c_m} \right)} \right]\end{array}$$

where $a$ ($a^{\dagger}$) and $c$ ($c^{\dagger}$) are the annihilation (creation) operators for resonant modes ${{{\mathrm{A}}}}_m$ and ${{{\mathrm{C}}}}_m$ in rings A and C, respectively, $\kappa _{{{{\mathrm{TB}}}}} = \kappa /T_{\mathrm{R}}$, $\eta _{{{{\mathrm{TB}}}}} = \kappa^{\prime}\gamma ^2/4T_{{{\mathrm{R}}}}$ for the weakly coupling case, and $\phi$ can be either $\pi$ or $0$, depending on what model we are going to study. $T_{{{\mathrm{R}}}} \equiv 2\pi /{{\Omega }}$. For the case of $\phi = \pi$, it corresponds to the diagram shown in Fig. 1c. The Hamiltonian can be rewritten under the rotating-wave approximation ($\kappa ,\kappa^{\prime},\gamma \ll 1$ and Ω ≪ *ω*_0_):

5 $$\begin{array}{l} \displaystyle H_{{{\mathrm{r}}}} = \mathop {\sum }\limits_m \left[ {\kappa _{{{{\mathrm{TB}}}}}\left( {e^{ - i\phi }a_m^{\dagger} a_{m + 1} + e^{i\phi }a_{m + 1}^{\dagger} a_m} \right) + \kappa _{{{{\mathrm{TB}}}}}\left( {c_m^{\dagger} c_{m + 1} + c_{m + 1}^{\dagger} c_m} \right)} \right.\\ \quad \,\,\quad \left. {+ \, \eta _{{{{\mathrm{TB}}}}}\left( {a_m^{\dagger} c_{m + 1} + c_{m + 1}^{\dagger} a_m} \right) + \eta _{{{{\mathrm{TB}}}}}\left( {e^{ - i\phi }c_m^{\dagger} a_{m + 1} + e^{i\phi }a_{m + 1}^{\dagger} c_m} \right)} \right]/2\end{array}$$

Equation (5) with $\phi = \pi$ describes a topological Hamiltonian of a one-dimensional pseudospin-$1/2$ lattice model (with the modes A and C denoting the spin-up and spin-down, respectively) along the synthetic frequency dimension as shown in Fig. 1c^32^. One notes that the Hamiltonian in Eq. (5) is only for an illustrative purpose. In the following, we proceed to study the quench dynamics and shall also show how the dynamical topological patterns emerge in a nontrivial way from simulations using a realistic model based on the setting in Fig. 1a.

### Results analysis

Now, we show the feasibility of directly measuring the bulk topology of the system by the quench dynamics process. The detailed simulation procedure is summarized in “Methods.” To this purpose, we first prepare the initial state of the system by injecting a monochromic light at the center frequency $\omega _{{{{\mathrm{C}}}},0}$ into the input external waveguide C. This source field has the temporal form with a normalized field amplitude $s$

6 $$E_0^{{{{\mathrm{C}}}},{{{\mathrm{in}}}}} = s\left\{ {\tanh \left[ {0.05\left( {t + t_{{{\mathrm{S}}}} - t_{{{\mathrm{O}}}}/2} \right)} \right] + \tanh \left[ {0.05\left( { - t_{{{\mathrm{O}}}}/2 - t} \right)} \right]} \right\}$$

where $t_{{{\mathrm{O}}}}$ is the turn-on/turn-off time and $t_{{{\mathrm{S}}}}$ is the pulse temporal duration. This choice of the input source only excites the mode ${{{\mathrm{C}}}}_{m = 0}$ in ring C. No mode in ring A is prepared at $t = 0$. Thus, the initial excitation of the ring system is fully polarized, giving an initial deep trivial state^30^. The modulations are then turned on at $t = 0$ in the time sequence diagram shown in Fig. 1d with the modulation time $t_{{{\mathrm{M}}}}$. Signals from output external waveguides are collected for further analysis in our simulations. The turning-on of the modulations makes the system be characterized by the nontrivial pseudospin-1/2 lattice model described in Fig. 1(c), and the quench dynamics is induced with the initial state evolving under the topological Hamiltonian of Eq. (5).

For the simulation, we set both ring A and ring C such that each contains 81 resonant modes ($m = - 40, - 39, \ldots ,40$). The parameters designed in the ring-resonator system in Fig. 1a are: $\kappa = 0.0025$, $\kappa^{\prime} = 0.2$, $\gamma = 0.1$, $\gamma^{\prime} = 0.003$, respectively. We also choose $t_{{{\mathrm{S}}}} = 1000\,T_{{{\mathrm{R}}}}$, $t_{{{\mathrm{O}}}} = 200\,T_{{{\mathrm{R}}}}$, and $t_{{{\mathrm{M}}}} = 15,000\,T_{{{\mathrm{R}}}}$.

**Fig. 2:**
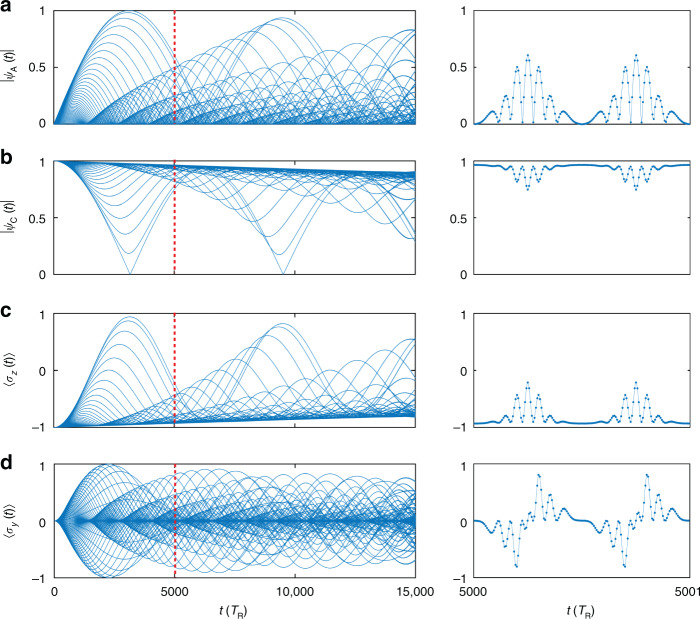
The electric field amplitudes and spin texture from simulations. **a**, **b** The normalized electric field amplitudes $\left| {\psi _{{{\mathrm{A}}}}} \right|$ and $\left| {\psi _{{{\mathrm{C}}}}} \right|$ versus discrete time collected from the output waveguides. One hundred and sixty data points are collected in one round-trip ($T_{{{\mathrm{R}}}}$). **c**, **d** The time evolution of normalized spin textures ($\left\langle {\sigma _z} \right\rangle$ and $\left\langle {\sigma _y} \right\rangle$). Right panels are the corresponding zoom-in plots, where data points are connected with lines, showing that $\left| {\psi _{{{\mathrm{A}}}}} \right|$, $\left| {\psi _{{{\mathrm{C}}}}} \right|$, $\left\langle {\sigma _z} \right\rangle$, and $\left\langle {\sigma _y} \right\rangle$ are evolving continuously along the time dimension

Signals are collected from $t = 0$ to $t = t_{{{\mathrm{M}}}}$ for all the frequency components $E_m^{{{{\mathrm{A}}}},{{{\mathrm{out}}}}}(t)$ and $E_m^{{{{\mathrm{C}}}},{{{\mathrm{out}}}}}(t)$ at both output waveguides. Therefore, the total electric field amplitudes of the signals, $\psi _{{{\mathrm{A}}}}(t)$ and $\psi _{{{\mathrm{C}}}}(t)$, can be retrieved by $\psi _{{{\mathrm{A}}}}\left( t \right) = \mathop {\sum }\nolimits_m E_m^{{{{\mathrm{A}}}},{{{\mathrm{out}}}}}(t)e^{ - i\omega _{{{{\mathrm{A}}}},m}t}$, and $\psi _{{{\mathrm{C}}}}\left( t \right) = \mathop {\sum }\nolimits_m E_m^{{{{\mathrm{C}}}},{{{\mathrm{out}}}}}(t)e^{ - i\omega _{{{{\mathrm{C}}}},m}t}$. We plot normalized $\left| {\psi _{{{\mathrm{A}}}}(t)} \right|$ and $\left| {\psi _{{{\mathrm{C}}}}(t)} \right|$ under the time evolution in Fig. 2a, b, respectively, which show nearly periodic patterns over the short time [see the zoom-in plots in both figures]. Nevertheless, the dynamics does not show the periodic stability over a long time, which is an evidence for the steady-state solution of the system^65^. With the collected signal, one can further construct the spin textures $\left\langle\sigma _z\left( t \right) \right\rangle= \left| {\psi _{{{\mathrm{A}}}}} \right|^2 - \left| {\psi _{{{\mathrm{C}}}}} \right|^2$ and $\left\langle\sigma _y\left( t \right)\right\rangle = - i\psi _{{{\mathrm{A}}}}^ \ast \cdot \psi _{{{\mathrm{C}}}}e^{i\Omega t/4} + i\psi _{{{\mathrm{C}}}}^ \ast e^{ - i\Omega t/4} \cdot \psi _A$, with which we shall show the essential prediction of this work that the two time fundamental scales emerge in the dynamics and the novel topological patterns are resulted (see also Supplementary Note 1 for details). Time evolution of normalized $\left\langle {\sigma _z(t)} \right\rangle$ and $\left\langle {\sigma _y(t)} \right\rangle$ are plotted in Fig. 2c, d. Note that the raw pseudospin dynamics characterized by $\left\langle {\sigma _z(t)} \right\rangle$ and $\left\langle {\sigma _y(t)} \right\rangle$ do not exhibit topological feature explicitly because the BIS, which characterizes the topological feature in the quench dynamic, is not explicitly shown in the plots in Fig. 2c, d. However, $\left\langle {\sigma _z(t)} \right\rangle$ and $\left\langle {\sigma _y(t)} \right\rangle$ actually contain the complete information as presented below.

### Topological quench dynamics in the synthetic frequency dimension

A novel observation is that two fundamental time scales emerge in the time evolution of the pseudospin polarization, denoted as the slow time variable $T$ and the fast time variable $\tau$, respectively. The real time reads $t = TT_{{{\mathrm{R}}}} + \tau$, with $T_{{{\mathrm{R}}}} = 2\pi /{{\Omega }}$. Thus, $T$ is the round-trip numbers, which is a discrete nonnegative integer $(T = 0,1,2, \ldots )$, and $\tau \in \left[ {0,T_{{{\mathrm{R}}}}} \right)$ is the round-trip time, which denotes the time within each round-trip. Note that for the synthetic dimension along the frequency axis of light, the round-trip time $\tau$ corresponds to the Bloch momentum $k_f$, i.e., the wave vector reciprocal to the frequency^65^. For studies modeling a static system, the transmission of light versus $\tau$ at the periodicity $T_{{{\mathrm{R}}}}$ can give the steady-state band structure of the synthetic lattice along the frequency axis of light^57,65^. However, in our present study, the topological quench dynamics is extracted from the two emergent time scales in the time dimension, of which $\tau$ mimics the *Bloch momentum* and $T$ denotes the *residue* time evolution of the state. Therefore, at each $T$, an individual band structure versus $\tau$ ($k_f$) can be retrieved, and then by varying $T$, the evolution of the band structure can be re-built, which leads to the dynamical pattern by using the field information only on $t$.

We therefore represent results $\left\langle {\sigma _z(t)} \right\rangle$ and $\left\langle {\sigma _y(t)} \right\rangle$ by defining $\left\langle {\sigma _z(T,\tau )} \right\rangle$ and $\left\langle {\sigma _y(T,\tau )} \right\rangle$, which give the quench dynamics at the Bloch momenta $k_f = \tau$ evolving over the discrete residue time $T$. The dynamical classification theory^30^ states that the quench dynamics exhibit nontrivial topology captured by the time-averaged spin texture in momentum space. Unlike the previous theory, here we define the averaged spin polarization $\overline {\left\langle {\sigma _z(T,\tau )} \right\rangle }$ and $\overline {\left\langle {\sigma _y(T,\tau )} \right\rangle }$ over the residue time $T$, given by

7 $$\overline {\langle {\sigma _{y,z}(T,\tau )}\rangle} = \frac{1}{{T + 1}}\mathop {\sum}\limits_{T^{\prime} = 0}^T {\langle { {\sigma _{y,z}(T^{\prime},\tau )} }\rangle }$$

**Fig. 3:**
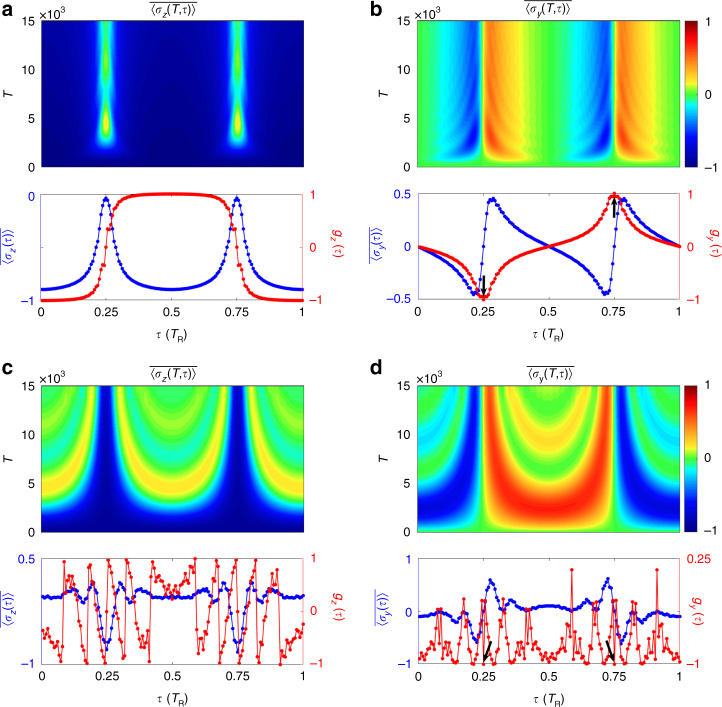
The time evolution of the normalized spin textures reconstructed by using two time scales ($\tau$ and $T$). **a**, **b** The evolution of averaged spin polarization $\overline {\langle {\sigma _{z,y}( {T,\tau })}\rangle }$, the overall spin polarization $\overline {\langle {\sigma _{z,y}( \tau)}\rangle }$, the dynamical spin texture $g_{z,y}(\tau )$, respectively, with $\phi = \pi$. **c**, **d** The evolution of averaged spin polarization $\overline{\langle {\sigma _{z,y}( {T,\tau })} \rangle}$, the overall spin polarization $\overline {\langle {\sigma _{z,y}( \tau)}\rangle }$, the dynamical spin texture $g_{z,y}(\tau )$, respectively, with $\phi = 0$. Black arrows point to values of $g_y(\tau _{1,2})$

We show $\overline {\langle{\sigma _{y,z}(T,\tau )}\rangle }$ and the overall spin polarization $\overline{\langle {\sigma _{y,z}(\tau )}\rangle } \equiv \mathop {{\lim }}\nolimits_{T \to \infty } \overline{\langle {\sigma _{y,z}(T,\tau )}\rangle }$ in Fig. 3a, b. The plots exhibit nontrivial dynamical pattern characterized via the two time scales $\tau$ and $T$. Firstly, the overall averaged polarizations vanish $\overline {\langle {\sigma_{y,z}(\tau)}\rangle} = 0$ at two special points with $\tau _1 = 0.25\,T_{{{\mathrm{R}}}}$ and $\tau _2 = 0.75\,T_{{{\mathrm{R}}}}$. Such two characteristic points are known as band inversion points in the 1D Brillouin zone^30^. Secondly, we define a new dynamical spin texture in the following way:

8 $$\vec g( \tau) = \left\{ {\begin{array}{l} {( {1/{{{\mathcal{N}}}}})\partial _\tau \overline{\langle { {\vec \sigma ( \tau)} }\rangle}, \,\tau = \tau _{1,2}} \\ {( {s/{{{\mathcal{N}}}}})\overline{\langle { {\vec \sigma ( \tau)} }\rangle} ,\,{\mathrm{other}}\,\tau \,{\mathrm{points}}} \end{array}} \right.$$

where ${{{\mathcal{N}}}}$ is the normalization factor, the derivative direction is chosen from the area in-between the two band inversion points to that out of them if $\tau$ is at band inversion points, and *s* = −1 ($+ 1$) if $\tau$ is in the region in-between (out of) the two band inversion points for other $\tau$ points. One finds that at the two band inversion points $g_z\left( {\tau _{1,2}} \right) = 0$, while $g_y\left( {\tau _1} \right) = - g_y\left( {\tau _2} \right) = - 1$ points in opposite directions [see Fig. 3b], giving a nonzero dynamical topological number, i.e., the zeroth Chern number $C_0 = [g_y\left( {\tau _2} \right) - g_y\left( {\tau _1} \right)]/2$ being a 0D invariant defined via the two band inversion points^30^ and equal to the 1D bulk topology of the post-quench Hamiltonian of the synthetic lattice constructed in Fig. 1c. This manifests the emergent dynamical correspondence between the 1D bulk topology of the equilibrium phase and 0D dynamical topology emerging in band inversion points. As a comparison, we consider the same system with the modulation phase $\phi = 0$, and show the numerical results in Fig. 3c, d. The emergent dynamical field $\vec g(\tau )$ exhibits no topological feature. In particular, $\vec g(\tau )$ is same at the two band inversion points [Fig. 3d], corresponding to the trivial case.

The above results of quench dynamics can be understood from the tight-binding model given in Eq. (5), which further takes the form in the momentum $k$-space

9 $$\begin{array}{l}H_k = \kappa _{{{{\mathrm{TB}}}}}\left( {a_k^{\dagger} a_ke^{ikd}e^{ - i\phi } + a_k^{\dagger} a_ke^{ - ikd}e^{i\phi } + c_k^{\dagger} c_ke^{ikd} + c_k^{\dagger} c_ke^{ - ikd}} \right)/2\\ \quad \quad \quad + \,\eta _{{{{\mathrm{TB}}}}}\left( {a_k^{\dagger} c_ke^{ikd} + a_k^{\dagger} c_ke^{ - ikd}e^{i\phi } + c_k^{\dagger} a_ke^{ - ikd} + c_k^{\dagger} a_ke^{ikd}e^{ - i\phi }} \right)/2\end{array}$$

with $d$ the lattice constant. For $\phi = \pi$, the above Hamiltonian gives a 1D topological phase known as AIII class insulator and is characterized by a 1D winding number^21,74^ (see also details in Supplementary Note 2). The dynamical topological number defined through $\vec g(\tau _{1,2})$ in quench dynamics precisely corresponds to the 1D winding number of the above Bloch Hamiltonian. On the other hand, for $\phi = 0$ the above Hamiltonian gives a 1D gapless spin–orbit coupled band with trivial topology (see comparison with calculations based on the tight-binding model in Supplementary Note 2).

We emphasize the highly nontrivial features of the topological quench dynamics, which provide the holographic characterization of the topological phase realized in the ring-resonator system, namely, the quench dynamics solely in the time dimension carries the complete information. The single variable, i.e., the time $t$, automatically splits into two fundamental time scales, mimicking the Bloch momenta $\tau$ of the topological band and the residue time evolution $T$ after quench, respectively, with which the bulk topology of the system is completely determined (Supplementary Movies 1). Specifically, the pseudospin dynamics averaged over the time scale $T$ manifest BIS structure depicted via $\tau$. The derivative of the $T$-averaged spin dynamics with respect to $\tau$ across BIS points determines the bulk topology through the bulk-surface duality. This result is in sharp contrast to the previous characterization of equilibrium topological phases through the nonequilibrium topological invariants^30–36^, which necessitates the information in both the time dimension and momentum space. On the other hand, this prediction also shows the novelty of classifying topological theory by the nonequilibrium dynamics, whose raw features are quite complicated and depend on system details (Fig. 2), but are actually classified by the underlying universal topological patterns (Fig. 3) through the characterization scheme given above (see Supplementary Movies 1).

### Topological quench dynamics with disorders in the phase modulator

Next, we consider the perturbation of topological quench dynamics from disorders in phase modulators. Such disorders in the phase modulation can be reflected in modulation strengths, $\kappa$ and $\kappa^{\prime}$. We consider that $\kappa$ and $\kappa^{\prime}$ undergo a random perturbation continuously, which is varying over time and can be described by $\kappa \left( t \right) = \kappa _0 \cdot f(t)$, $\kappa^{\prime}\left( t \right) = \kappa^{\prime}_0 \cdot f(t)$, where $\kappa _0 = 0.0025$ and $\kappa^{\prime}_0 = 0.2$. Here $f\left( t \right) = 1 + \delta \cdot r(t)$ is the disorder function, where $r(t)$ is a time-varying random function with a range $[ - 0.5,0.5]$ and $\delta$ represents the disorder intensity.

**Fig. 4:**
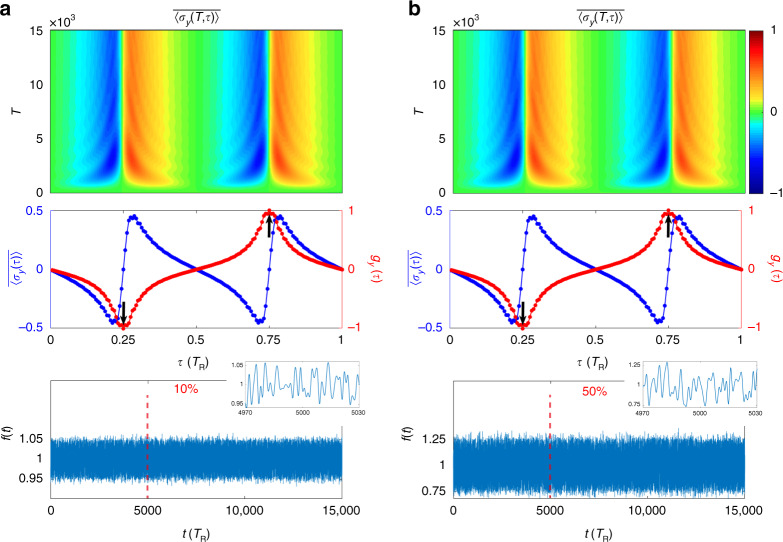
The time evolution of the normalized spin textures with disorders in the phase modulator. The evolution of averaged spin polarization $\overline {\langle {\sigma _y( {T,\tau })}\rangle }$, the overall spin polarization $\overline {\langle {\sigma _y( \tau)}\rangle }$, the dynamical spin texture $g_y(\tau )$, respectively, with parameters for Fig. 3a, b and disorder function $f(t)$ in $\kappa (t)$ and $\kappa^{\prime}(t)$ with $\delta = 10\%$ (**a**) and $50\%$ (**b**), respectively. Black arrows point to values of $g_y(\tau _{1,2})$

Simulations are performed with parameters for Fig. 3a, b and $\delta = 10\%$ and $50\%$, respectively, and results of $\overline {\left\langle {\sigma _y\left( {T,\tau } \right)} \right\rangle }$ together with $\overline {\langle {{\sigma _y( \tau)} }\rangle}$ and $g_y(\tau )$ are plotted in Fig. 4, with the corresponding $f(t)$. Compared to the dynamical pattern in Fig. 3(b), evolutions of $\overline {\left\langle {\sigma _y\left( {T,\tau } \right)} \right\rangle }$ with different disorder $\delta$ show relatively similar profiles. The averaged spin-polarization pattern and the nontrivial dynamical spin texture preserve when phase modulators include temporal disorders. This result can be understood since the temporal disorder in $\kappa$ and $\kappa^{\prime}$ does not break the symmetry feature in the Hamiltonian in Eq. (9), and hence the bulk topology of the Hamiltonian preserves.

### Topological quench dynamics with disorders in the input source

In the simulation for Fig. 3a, b, we prepare the initial state of the system by injecting a monochromic light at the center frequency $\omega _{{{{\mathrm{C}}}},0}$. Here, we consider the injected light has a disorder in both intensities and phases for all frequency modes. Such disorder in the input source can be described by

10 $$\left\{ {\begin{array}{l}\displaystyle {E_0^{{{{\mathrm{C,in}}}}}} =\, {(1 - \delta \cdot R) \cdot s \cdot \{\tanh [0.05(t + t_{\mathrm{S}}-t_{{{\mathrm{O}}}}/2)] + \tanh [0.05( - t_{{{\mathrm{O}}}}/2 - t)]\}} \\ {E_{m \ne 0}^{{{{\mathrm{C,in}}}}}} =\,{\delta \cdot R \cdot e^{i2\pi R},\quad 0 \le t \le t_{\mathrm{S}}} \end{array}} \right.$$

where $\delta$ is the disorder intensity and $R$ gives a random number in the range $[ - 0.5,0.5]$.

**Fig. 5:**
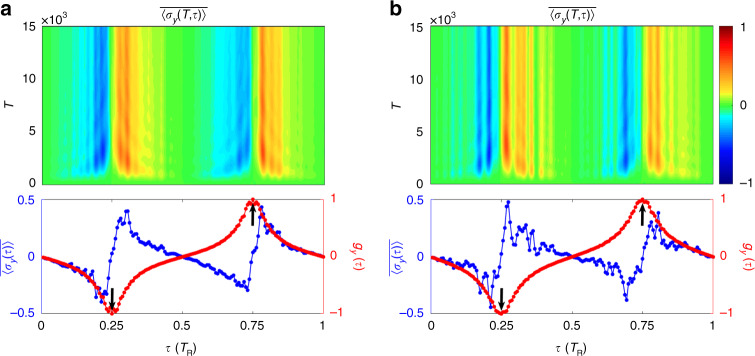
The time evolution of the normalized spin textures with disorders in the input source. The evolution of averaged spin polarization $\overline {\langle {\sigma _y( {T,\tau })}\rangle}$, the overall spin polarization $\overline{\langle {\sigma _y( \tau)}\rangle }$, the dynamical spin texture $g_y(\tau )$, respectively, with parameters for Fig. 3a, b and disorder in the input source in Eq. (10) with $\delta = 5\%$ (**a**) and $10\%$ (**b**), respectively. Black arrows point to values of $g_y(\tau _{1,2})$

We perform simulations with the same parameters for Fig. 3a, b, and the input source in Eq. (10) with $\delta = 5\%$ and $10\%$, respectively. The corresponding evolutions of $\overline {\left\langle {\sigma _y\left( {T,\tau } \right)} \right\rangle }$ together with $\overline{\langle{\sigma _y( \tau)}\rangle}$ and $g_y(\tau )$ are plotted in Fig. 5. Although the disorder in the input source affects more largely the topological quench dynamics for the case with larger $\delta$, the overall spin polarization in $\overline{\langle { {\sigma _y( \tau)} }\rangle}$ as well as the nontrivial dynamical spin texture $g_y(\tau )$ still capture the topological feature of the studied system.

### Experimental feasibility

The quench dynamics is induced by initializing a deep trivial phase for the topological Hamiltonian, which in principle has high experimental feasibility in comparison with the currently achieved band structure measurement for the resonator ring systems^65,66^. In the present study, we do not need to prepare the initial system to be in the eigenstates of the Hamiltonian, nor to scan the frequency to match the band energies, which are, however, required and were the major challenges for the conventional band mapping techniques. This essential difference makes the present quench study be of high feasibility.

Further, the proposed ring-resonator system can be achieved in both fiber-based platforms^57,65,66^ and on-chip lithium niobate photonic designs^75,76^, where the parameters in the system can be realized in experiments. In both systems, the conversion efficiency of the electro-optic modulators can reach up to $\sim\!2\%$^76–79^, which is sufficient for the proposed system here. The quality factor for the ring is potentially possible at the order of $\sim\!10^7 - 10^8$ with an amplifier for compensation in fiber rings^57,65^ or the state-of-art integrated lithium niobate technology^80^. The frequency shift in rings A and C is feasible by slightly changing the length of one ring given the case that we consider frequency components in the optical regime and modulations at the order of MHz to GHz. In both experimental platforms, light is injected through the coupling waveguide into rings, and signals are collected from two drop-off ports. With the interference of fields from two output waveguides [see Fig. 1a], the spin textures are then collected for obtaining dynamical spin texture through further digital data analysis. Therefore, our proposal provides an experimental feasible platform for measuring the quench dynamics and the topological invariants directly from the temporal optical signal in a ring resonator, which can lead to significant simplification for performing dynamical characterization of topological quantum phases in different synthetic models.

## Discussions

In summary, we have proposed the topological holographic quench dynamics by investigating the far-from-equilibrium topological physics in a 1D spinful lattice model synthesized in the frequency dimension of ring resonators. In particular, we showed that the quench dynamics in time dimensions is novelly characterized with two emergent fundamental time scales, with one mimicking the Bloch momentum of the lattice and the other characterizing the residue time evolution. From this characterization, the quench dynamics carry the complete topological information and exhibit universal dynamical topological patterns that correspond to the equilibrium topological phase of the spin model. The topological quench dynamics is robust against disorders and of high feasibility in experimental realization. We note that the approach proposed in this work is generic, and the study can be readily extended to topological phases in synthetic high-dimensions, e.g., to the 2D Haldane model^71^. In that case, we expect that the multiple fundamental time scales would emerge in the holographic quench dynamics, with some mimicking the high-dimensional Bloch momenta and the remaining characterizing the residue time evolution, for which the complex quench dynamics can be classified by the exotic dynamical topology and has a profound connection to the bulk topology of the post-quench Hamiltonian through the dynamical bulk-surface duality. This work showed a unique way to study the holographic far-from-equilibrium dynamics, with the rich and complex topological physics being encoded in only the single variable, i.e., the time evolution, and shall provide the insight into the exploration of the high-dimensional topological phases with quench dynamics in the synthetic photonic crystals.

## Materials and methods

### Simulation method

We perform simulations using the realistic model based on the setting in Fig. 1a. The simulation has been used to successfully describe the dynamics of the ring-based system in the synthetic space and is discussed in detail in refs. ^52,67,71^. Here, we briefly summarize the procedure. The electric field inside the waveguide is^81^

11 $$E\left( {t,r_ \bot ,x} \right) = \mathop {\sum }\limits_m {{{\mathcal{E}}}}_m\left( {t,x} \right)E_m\left( {r_ \bot } \right)e^{i\omega^{\prime}_mt}$$

where $x$ is the propagation direction along the waveguide that composes the ring resonator, $r_ \bot$ is the perpendicular directions of $x$, $\omega^{\prime}_m$ is either $\omega _{{{{\mathrm{A}}}},m}$ or $\omega _{{{{\mathrm{C}}}},m}$, $E_m(r_ \bot )$ is the modal profile for the ring A or C as well as the auxiliary ring B, and ${{{\mathcal{E}}}}_m(t,x)$ is the associated modal amplitude in different rings. Under the slowly varying envelope approximation, Eq. (11) satisfies the wave equation:

12 $$\left[ {\frac{\partial }{{\partial x}} + i\beta \left( {\omega ^{\prime}_m} \right)} \right]{{{\mathcal{E}}}}_m + \frac{{n_{{{\mathrm{g}}}}}}{c}\frac{\partial }{{\partial t}}{{{\mathcal{E}}}}_m = 0$$

where $\beta$ is the wavevector. The ring has the periodic boundary condition ${{{\mathcal{E}}}}_m\left( {t,x + L} \right) = {{{\mathcal{E}}}}_m(t,x)$ for rings A and C, and ${{{\mathcal{E}}}}_m\left( {t,x + 4L} \right) = {{{\mathcal{E}}}}_m(t,x)$ for ring B.

When the light passes through the phase modulation inside the waveguide, the field undergoes dynamic modulation and modal amplitudes obey^82^:

13 $$\begin{array}{ll}{{{\mathcal{E}}}}_m^{{{{\mathrm{A}}}}({{{\mathrm{C}}}})}\left( {t^ + ,x_{0}^{{{{\mathrm{a}}}}({{{\mathrm{c}}}})}} \right) = \,J_0\left( \kappa \right){{{\mathcal{E}}}}_m^{{{{\mathrm{A}}}}({{{\mathrm{C}}}})}\left( {t^ - ,x_0^{{{{\mathrm{a}}}}({{{\mathrm{c}}}})}} \right)\\ \qquad\qquad\qquad\qquad+ \,\, J_1\left( \kappa \right){{{\mathcal{E}}}}_{m - 1}^{{{{\mathrm{A}}}}({{{\mathrm{C}}}})}\left( {t^ - ,x_0^{{{{\mathrm{a}}}}({{{\mathrm{c}}}})}} \right)e^{i\phi _{{{{\mathrm{A}}}}({{{\mathrm{C}}}})}}\\ \qquad\qquad\qquad\qquad -\,\, J_1\left( \kappa \right){{{\mathcal{E}}}}_{m + 1}^{{{{\mathrm{A}}}}({{{\mathrm{C}}}})}\left( {t^ - ,x_0^{{{{\mathrm{a}}}}({{{\mathrm{c}}}})}} \right)e^{ - i\phi _{{{{\mathrm{A}}}}({{{\mathrm{C}}}})}}\end{array}$$

where $t^ \pm = t + 0^ \pm$, $x_0^{{{{\mathrm{a}}}}({{{\mathrm{c}}}})}$ represents the position of the modulator in the ring A or C in Fig. 1a, and $J_0$ and $J_1$ are the zeroth- and first-order Bessel functions, respectively. Here we take the first-order approximation and only consider the nearest-neighbor couplings, which turns out to be fine in this model and also in other works for weak modulations^52,67,71^. Similarly, dynamic modulations on both PM BA and PM BC at positions $x_0^{{{{\mathrm{ba}}}}}$ and $x_0^{{{{\mathrm{bc}}}}}$, respectively, are described by the following equations:

14 $$\begin{array}{l}\displaystyle{{{\mathcal{E}}}}_m^{{{{\mathrm{BA}}}}\left( {{{{\mathrm{BC}}}}} \right)}\left( {t^ + ,x_0^{{{{\mathrm{ba}}}}\left( {{{{\mathrm{bc}}}}} \right)}} \right) = J_0\left( {\kappa^{\prime}} \right){{{\mathcal{E}}}}_m^{{{{\mathrm{BA}}}}\left( {{{{\mathrm{BC}}}}} \right)}\left( {t^ - ,x_0^{{{{\mathrm{ba}}}}\left( {{{{\mathrm{bc}}}}} \right)}} \right) - J_1\left( {\kappa^{\prime}} \right){{{\mathcal{E}}}}_{m + 1}^{{{{\mathrm{BC}}}}\left( {{{{\mathrm{BA}}}}} \right)}\left( {t^ - ,x_0^{{{{\mathrm{ba}}}}\left( {{{{\mathrm{bc}}}}} \right)}} \right)e^{ - i\phi _{{{{\mathrm{BA}}}}\left( {{{{\mathrm{BC}}}}} \right)}}\\ \,{{{\mathcal{E}}}}_m^{{{{\mathrm{BA}}}}\left( {{{{\mathrm{BC}}}}} \right)}\left( {t^ + ,x_0^{{{{\mathrm{bc}}}}\left( {{{{\mathrm{ba}}}}} \right)}} \right) = J_0\left( {\kappa ^{\prime}} \right){{{\mathcal{E}}}}_m^{{{{\mathrm{BA}}}}\left( {{{{\mathrm{BC}}}}} \right)}\left( {t^ - ,x_0^{{{{\mathrm{bc}}}}\left( {{{{\mathrm{ba}}}}} \right)}} \right) + J_1\left( {\kappa^{\prime}} \right){{{\mathcal{E}}}}_{m - 1}^{{{{\mathrm{BC}}}}\left( {{{{\mathrm{BA}}}}} \right)}\left( {t^ - ,x_0^{{{{\mathrm{bc}}}}\left( {{{{\mathrm{ba}}}}} \right)}} \right)e^{i\phi _{{{{\mathrm{BC}}}}\left( {{{{\mathrm{BA}}}}} \right)}}\end{array}$$

We keep only the field components that have frequencies resonant in either ring A or C, but omit components that are nonresonant in all three rings because the energies of these nonresonant modes diminish quickly after circulating several roundtrips inside the ring. Equation (13) and (14) reflect transmission coefficients in Eqs. (1) and (2), respectively, i.e., $E\left( {t^ + ,r_ \bot ,x_0} \right) = TE\left( {t^ - ,r_ \bot ,x_0} \right)$.

The coupling in Eq. (3) between fields in rings A and B through the evanescent wave at corresponding positions in Fig. 1a can be described by:

15 $${{{\mathcal{E}}}}_m^{{{{\mathrm{A}}}}\left( {{{{\mathrm{BA}}}}} \right)}\left( {t^ + ,x_1^{{{{\mathrm{a}}}}\left( {{{{\mathrm{ba}}}}} \right)}} \right) = \sqrt {1 - \gamma ^2} {{{\mathcal{E}}}}_m^{{{{\mathrm{A}}}}\left( {{{{\mathrm{BA}}}}} \right)}\left( {t^ - ,x_1^{{{{\mathrm{a}}}}\left( {{{{\mathrm{ba}}}}} \right)}} \right) - i\gamma {{{\mathcal{E}}}}_m^{{{{\mathrm{BA}}}}\left( {{{\mathrm{A}}}} \right)}\left( {t^ - ,x_1^{{{{\mathrm{ba}}}}\left( {{{{\mathrm{a}}}}} \right)}} \right)$$

The coupling between fields in rings C and B is similarly described.

In simulations, the four external waveguides coupling rings A and C, as shown in Fig. 1a, are applied to input the source fields (which can also be decomposed to the frequency component $E_m^{{{{\mathrm{A}}}},{{{\mathrm{in}}}}}$ and $E_m^{{{{\mathrm{C}}}},{{{\mathrm{in}}}}}$) and detect the output signal ($E_m^{{{{\mathrm{A}}}},{{{\mathrm{out}}}}}$ and $E_m^{{{{\mathrm{C}}}},{{{\mathrm{out}}}}}$). The input/output coupling between the waveguide and the ring is also described by the similar Eq. (15) with the coupling strength $\gamma ^{\prime}$^71^.

## Supplementary information

Supplementary Information

Cartoon Movie

## Data Availability

All data in the paper and the Supplementary materials are available.
