# Supplementary material for: Topological holographic quench dynamics in a synthetic frequency dimension
Source: Light Sci Appl. 2021 Oct 7;10:209. doi: 10.1038/s41377-021-00646-y (PMC8497532; doi:10.1038/s41377-021-00646-y)
Supplement: Supplementary file 1 — Supplementary Information [file 41377_2021_646_MOESM1_ESM.pdf]

Supplementary Information for: Topological holographic quench dynamics in a synthetic frequency dimension

Danying Yu[1], Bo Peng[1], Xianfeng Chen[1,3,4,5], Xiong-Jun Liu[2,6,†], and Luqi Yuan[1,*]

[1]*State Key Laboratory of Advanced Optical Communication Systems and Networks, School of Physics and Astronomy, Shanghai Jiao Tong University, Shanghai 200240, China*

[2]*International Center for Quantum Materials and School of Physics, Peking University, Beijing 100871, China*

[3]*Shanghai Research Center for Quantum Sciences, Shanghai 201315, China*

[4]*Jinan Institute of Quantum Technology, Jinan 250101, China*

[5]*Collaborative Innovation Center of Light Manipulations and Applications, Shandong Normal University, Jinan 250358, China*

[6]*Shenzhen Institute for Quantum Science and Engineering, Southern University of Science and Technology, Shenzhen 518055, China*

*Corresponding authors: *yuanluqi@sjtu.edu.cn; †xiongjunliu@pku.edu.cn*

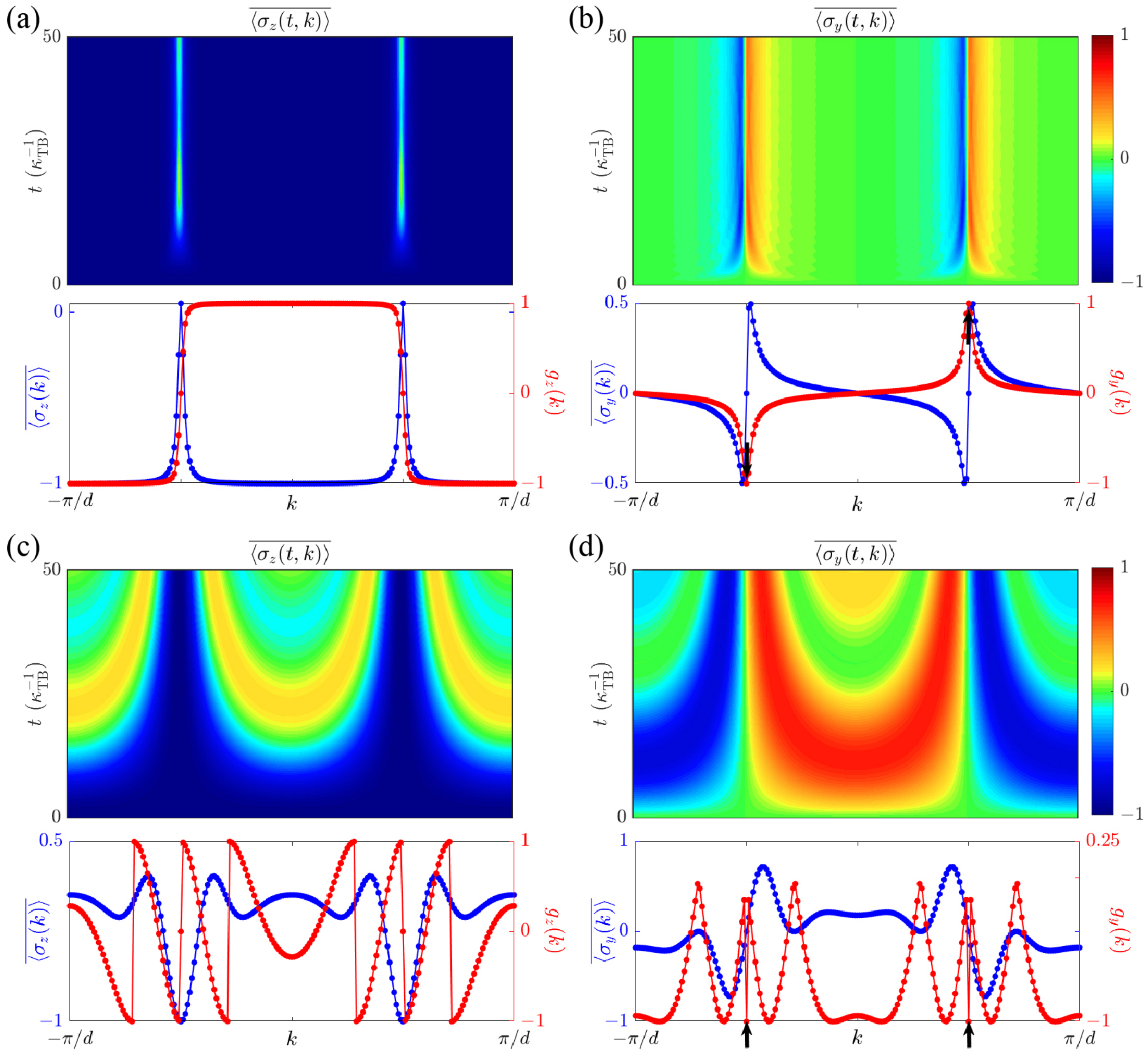

Fig. S1: Numerical simulation results of spin textures in the k-space. (a) and (b) The evolution of averaged spin-polarization $\overline{\langle\sigma_{z,y}(t,k)\rangle}$, the overall spin-polarization $\overline{\langle\sigma_{z,y}(k)\rangle}$, the dynamical spin texture $g_{z,y}(k)$, respectively, with $\phi=\pi$. (c) and (d) The evolution of averaged spin-polarization $\overline{\langle\sigma_{z,y}(t,k)\rangle}$, the overall spin-polarization $\overline{\langle\sigma_{z,y}(k)\rangle}$, the dynamical spin texture $g_{z,y}(k)$, respectively, with $\phi=0$. Black arrows point to values of $g_y(k=\pm\pi/2d)$

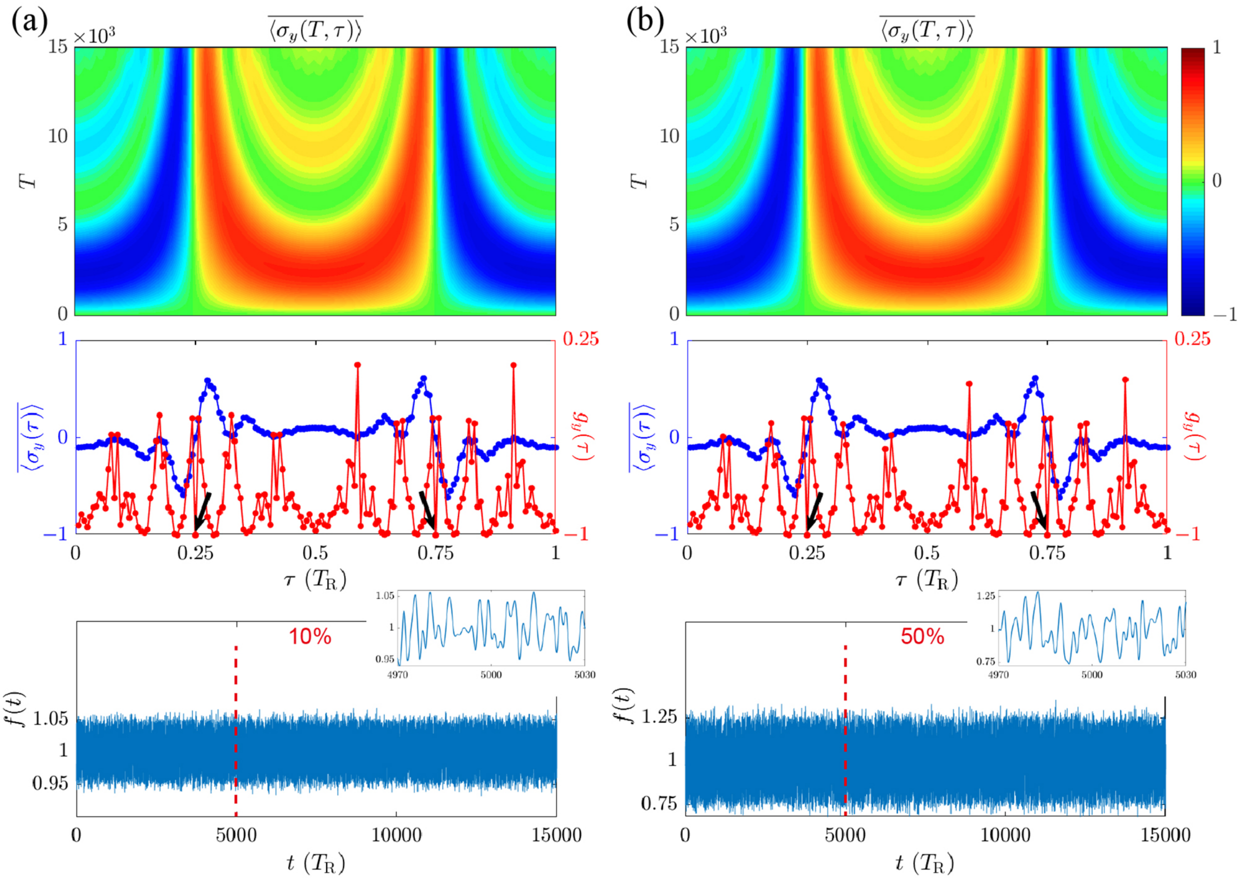

Fig. S2: The evolution of averaged spin-polarization $\overline{\langle\sigma_y(T,\tau)\rangle}$, the overall spin-polarization $\overline{\langle\sigma_y(\tau)\rangle}$, the dynamical spin texture $g_y(\tau)$ under the trivial condition, with same parameters for Fig. 3(c) and 3(d) and disorder function $f(t)$ in $\kappa(t)$ and $\kappa'(t)$ with $\delta = 10\%$(a) and 50%(b), respectively. Black arrows point to values of $g_y(\tau_{1,2})$.

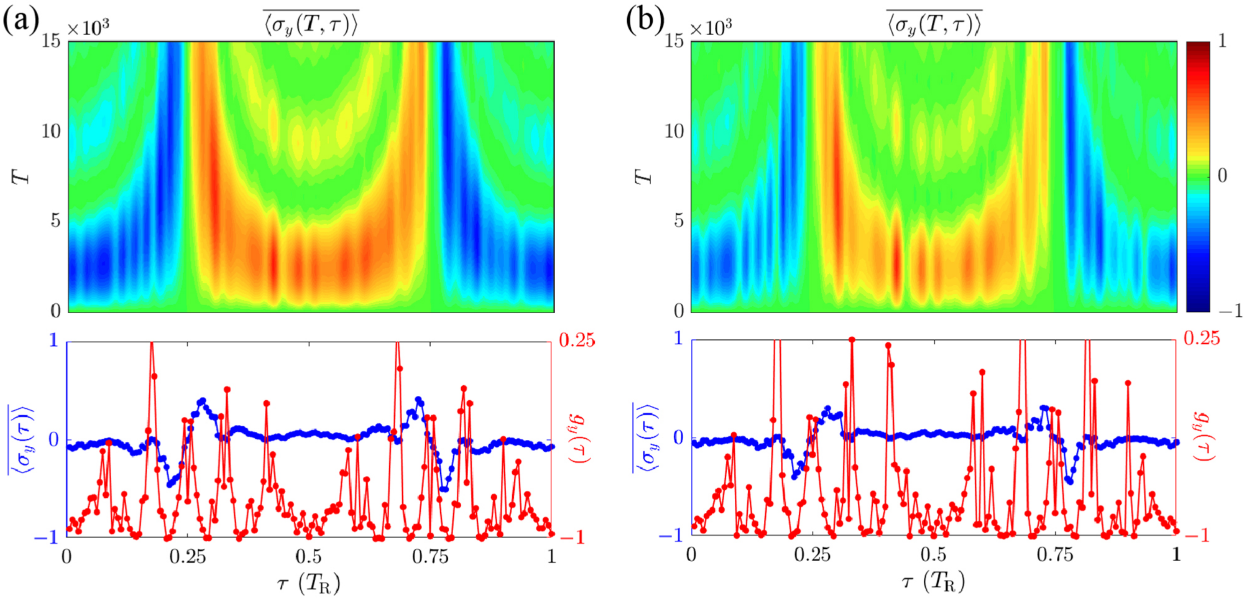

Fig. S3: The evolution of averaged spin-polarization $\overline{\langle\sigma_y(T,\tau)\rangle}$, the overall spin-polarization $\overline{\langle\sigma_y(\tau)\rangle}$, the dynamical spin texture $g_y(\tau)$ under the trivial condition, with parameters for Figs. 3(c) and 3(d) and disorder in the input source in Eq. (10) in the main text with $\delta = 5\%$(a) and 10%(b), respectively.

I. SUPPLEMENTARY NOTE 1 - RELATION BETWEEN FIELDS AND SPIN TEXTURES

Signals collected from output waveguide in Fig. 1(a) in the main text are $\psi_{\mathrm{A}}(t)$ and $\psi_{\mathrm{C}}(t)$, respectively. Spin textures of the one-dimensional pseudo-spin lattice in Fig. 1(c) in the main text include $\langle\sigma_z\rangle$ and $\langle\sigma_y\rangle$, respectively. The relation between spin textures $\langle\sigma_z\rangle$, $\langle\sigma_y\rangle$ and signals ψ_{A}, ψ_{C} satisfies:

$$\langle\sigma_z\rangle \equiv \left(\psi_{\mathrm{A}}^*, \psi_{\mathrm{C}}^* e^{-i\Omega t/4}\right)\begin{pmatrix}1 & 0\\ 0 & -1\end{pmatrix}\begin{pmatrix}\psi_{\mathrm{A}}\\ \psi_{\mathrm{C}} e^{i\Omega t/4}\end{pmatrix} = |\psi_{\mathrm{A}}|^2 - |\psi_{\mathrm{C}}|^2 \tag{S1}$$

$$\begin{aligned}\langle\sigma_y\rangle &\equiv \left(\psi_{\mathrm{A}}^*, \psi_{\mathrm{C}}^* e^{-i\Omega t/4}\right)\begin{pmatrix}0 & -i\\ i & 0\end{pmatrix}\begin{pmatrix}\psi_{\mathrm{A}}\\ \psi_{\mathrm{C}} e^{i\Omega t/4}\end{pmatrix}\\ &= -i\psi_{\mathrm{A}}^* \cdot \psi_{\mathrm{C}} e^{i\Omega t/4} + i\psi_{\mathrm{C}}^* e^{-i\Omega t/4} \cdot \psi_{\mathrm{A}}\end{aligned} \tag{S2}$$

Here the extra coefficient $e^{\pm i\Omega t/4}$ is from the frequency offset between rings A and C. It is obvious that Eqs. (S1) and (S2) can be obtained by subtraction of intensities of two optical signals and interference between two optical signals, respectively.

II. SUPPLEMENTARY NOTE 2 - COMPARISON WITH NUMERICAL RESULTS FROM TIGHT-BINDING MODELS

In this section, we show the numerical simulations of the corresponding spin textures by numerically solving the tight-binding model in Eq. (5) in the main text with $\phi = \pi$ or 0, respectively. The Hamiltonian can be re-written in the k-space:

$$\begin{aligned}H_k =& \kappa_{\mathrm{TB}}(a_k^\dagger a_k e^{ikd} e^{-i\phi} + a_k^\dagger a_k e^{-ikd} e^{i\phi} + c_k^\dagger c_k e^{ikd} + c_k^\dagger c_k e^{-ikd})/2\\ &+ \eta_{\mathrm{TB}}(a_k^\dagger c_k e^{ikd} + a_k^\dagger c_k e^{-ikd} e^{i\phi} + c_k^\dagger a_k e^{-ikd} + c_k^\dagger a_k e^{ikd} e^{-i\phi})/2\end{aligned} \tag{S3}$$

where d is the lattice constant. One can rewrite Eq. (S3) in the momentum space $H_k = -\kappa_{\mathrm{TB}}\cos(kd)\sigma_z - \eta_{\mathrm{TB}}\sin(kd)\sigma_y$ when $\phi = \pi$, which gives the 1D AIII class topological insulator with 1D winding number [1, 2], and $H_k = \kappa_{\mathrm{TB}}\cos(kd)I + \eta_{\mathrm{TB}}\cos(kd)\sigma_x$ when $\phi = 0$, which corresponds to a trivial phase. Here I is the unit matrix.

The Hamiltonian Eq. (S3) in the topological regime ($\phi = \pi$) and in quasi-momentum space reads $H_k = h_z(k)\sigma_z + h_y(k)\sigma_y$, where $h_z(k) = -\kappa_{\mathrm{TB}}\cos(kd)$ and $h_y(k) = -\eta_{\mathrm{TB}}\sin(kd)$.

The 1D winding number is simply characterized by the number of times that the unit vector $\bar{\mathbf{h}}(k) = (h_z(k), h_y(k))/|h(k)|$ covers the unit circle in the y-z plane when k runs over from 0 to 2π. This 1D winding number is further given by the quantities of $\bar{h}_y(k)$ on the band inversion points where $\bar{h}_z(k) = 0$ [3]. For example, if there are two band inversion points with $\bar{h}_z = 0$, and then the system is topologically nontrivial if $\bar{h}_y$ takes opposite values on the two band inversion points, defining a nonzero C_0. The dynamical spin texture $g_y(k) = \bar{h}_y(k)$ on two band inversion points, hence determines the topology of the post-quench system. Note that, in a model with the synthetic frequency dimension, the Bloch momenta k refers to the fast time variable τ, as illustrated in the main text.

The spin textures of the system can then be simulated with initial condition $\langle\sigma_z\rangle = -1, \langle\sigma_y\rangle = 0, \langle\sigma_x\rangle = 0$, and $\eta_{\mathrm{TB}} = 0.05\kappa_{\mathrm{TB}}$. The simulation results of $\langle\sigma_z(t,k)\rangle$ for $\phi = \pi$ and 0 are obtained, and corresponding $\overline{\langle\sigma_{z,y}(t,k)\rangle}$, $\overline{\langle\sigma_{z,y}(k)\rangle}$, and $g_{z,y}(k)$ can be defined in the same procedure as that in the main text and then be plotted in Fig. S1. One can notice that these numerical results give the non-trivial feature for $\phi = \pi$ and trivial feature for $\phi = 0$, respectively [3]. Comparing both results in Fig. 3 in the main text and Fig. S1, one finds the consistency in the quench dynamics which gives the same evidences for either non-trivial or trivial case.

III. SUPPLEMENTARY NOTE 3 - COMPARISONS WITH TRIVIAL CASES WITH DISORDERS

Quench dynamics with disorders in the phase modulators under the trivial case. In the main text, we have shown $\overline{\langle\sigma_y(T,\tau)\rangle}$ together with $\overline{\langle\sigma_y(\tau)\rangle}$ and $g_y(\tau)$ in the nontrivial case under the perturbation from the disorder in phase modulators. Here, we show the corresponding trivial case by setting $\phi = 0$ and using other parameters the same as those for generating Fig. 4 in the main text. In Fig. S2, evolutions of $\overline{\langle\sigma_y(T,\tau)\rangle}$ in the trivial case under different disorder δ show similar pattern compared with Fig. 3(d) in the main text. The averaged spin-polarization pattern and the dynamical spin texture also exhibit similar profiles compared with Fig. 3(d) in the main text under the trivial case.

Quench dynamics with disorders in the input source under the trivial case. In the main text, we plot $\overline{\langle\sigma_y(T,\tau)\rangle}$ together with $\overline{\langle\sigma_y(\tau)\rangle}$ and $g_y(\tau)$ under the perturbation from disorder in the input source in Fig. 5 for the nontrivial case. Here, we give the corresponding

simulation under the trivial case by setting $\phi = 0$. In Fig. S3, the disorder in the input source affects the pattern of $\overline{\langle\sigma_y(T,\tau)\rangle}$ with larger δ, but $\overline{\langle\sigma_y(\tau)\rangle}$ and $g_y(\tau)$ still give the similar profiles compared with Fig. 3(d) in the main text.

SUPPLEMENTARY REFERENCES

---

[1] Song, B et al. Observation of symmetry-protected topological band with ultracold fermions. *Science Advances* **4**, eaao4748 (2018).

[2] Liu, X. J., Liu, Z. X. & Cheng, M. Manipulating Topological Edge Spins in a One-Dimensional Optical Lattice. *Physical Review Letters* **110**, 076401 (2013).

[3] Zhang, L. et al. Dynamical classification of topological quantum phases. *Science Bulletin* **63**, 1385-1391 (2018).
